# Normative penile anthropometry in term newborns in Kumasi, Ghana: a cross-sectional prospective study

**DOI:** 10.1186/s13633-017-0042-1

**Published:** 2017-01-26

**Authors:** Serwah Bonsu Asafo-Agyei, Emmanuel Ameyaw, Jean-Pierre Chanoine, Samuel Blay Nguah

**Affiliations:** 1 Department of Child Health, Komfo Anokye Teaching Hospital, Kumasi, Ghana; 2 Endocrinology and Diabetes Unit, British Columbia’s Children’s Hospital, University of British Columbia, Vancouver, BC Canada

**Keywords:** Penile length, Penile width, Genital size, Ghana, Micropenis, Macropenis

## Abstract

**Background:**

Genital measurements are a useful adjunct in the early detection of various endocrine conditions including hypopituitarism and disorders of sexual differentiation. Standards for genital sizes have been published but racial/ethnic differences exist. This study was done to establish norms for genital sizes in term Ghanaian male newborns.

**Methods:**

This was a cross-sectional study of all apparently well full-term newborns of postnatal age < 48 h and birth weight between 2.5 and 4.0 kg delivered at Komfo Anokye Teaching Hospital within the study period. Anthropometric and genital parameters were documented for study subjects as well as parental socio-demographic indices.

**Results:**

A total of 644 male newborns were recruited between May and September 2014. The mean penile length (MPL) was 3.3 ± 0.5 cm and the mean penile width (MPW) was 1.05 ± 0.1 cm. An inverse relationship was found between maternal age and MPL (correlation coefficient −0.062, 95% CI −0.121 to −0.002; *p* = 0.04). MPL was also significantly different (*p* = 0.04) by mode of delivery, with babies delivered by caesarean section having the lowest MPL. MPL correlated positively with both gestational age (*p* = 0.04) and birth length (*p* < 0.001), while MPW correlated proportionally with birth weight and length (*p* < 0.001 for both).

**Conclusions:**

Using the conventional definition of micropenis as stretched penile length (SPL) < 2.5 standard deviation (SD) below the mean and macropenis as an SPL > 2.5 SD, a Ghanaian term newborn may warrant investigation if he has an MPL < 2.1 cm or > 4.4 cm.

**Electronic supplementary material:**

The online version of this article (doi:10.1186/s13633-017-0042-1) contains supplementary material, which is available to authorized users.

## Background

Penile size is a reflection of the adequacy of exposure of the male fetus to androgens, right from hypothalamic/pituitary gonadotropins down to testicular androgens. Normative standards of penile sizes are thus useful for the diagnosis of genital/endocrine abnormalities [1]. Penile length abnormalities consist of micropenis and macropenis (‘large penis’). A micropenis is an abnormally small penis with a normal configuration [2]. Conventionally, micropenis has been defined as a stretched penile length (SPL) < 2.5 standard deviation (SD) below the mean for age with normal function and structure. Likewise, an SPL > 2.5 SD is considered as a macropenis [2, 3].

Micropenis is an important sign in neonates, since it may be the only clue to the diagnosis of hypopituitarism, a potentially lethal but treatable condition [4]. It may also raise the clinical suspicion concerning the possibility of associated hypoglycaemia, a life-threatening metabolic emergency associated with hypopituitarism or isolated growth hormone deficiency [5]. Micropenis may also reflect an impairment specific to the hypothalamo-pituitary-gonadal axis, including isolated hypogonadism, poorly functioning, dysgenetic testicular tissue with malignant potential or partial androgen insensitivity [6, 7]. Early diagnosis of micropenis is important, because it allows for various treatment options to be implemented early [8].

Conversely, a possibly large penis with hyperpigmentation of the scrotum alerts the physician to the possibility of congenital adrenal hyperplasia (CAH), a condition that can be fatal if not identified [9]. This is especially crucial in resource limited settings where newborn screening for CAH is not routinely done, as newborn males with CAH usually don’t have other signs of androgen excess and often die undiagnosed from salt wasting crisis [10]. Macropenis has also been associated with rare syndromes like auriculo-condylar syndrome and caudal dysplasia sequence [11, 12].

Genital anthropometric measurements also help to avoid unwarranted investigations. It is useful in differentiating micropenis from an entirely normal ‘inconspicuous’ penis (concealed penis) [13].

Normative genital anthropometric data for healthy newborns exist and were mainly derived from Caucasian and Asian infants [3, 6, 14–16]. However, this may not be applicable to African infants. Indeed, previous studies have reported racial/ethnic differences in newborn penile sizes [1, 17, 18]. Some studies have even demonstrated significant differences in the same ethnic group with the passage of time and improvement in national economic conditions [19]. Recent studies from various parts of the world have aimed at establishing genital anthropometric norms representing their own populations [20–24]. No such comparable published data exist for Ghana. Hence, this study aimed at establishing the reference ranges for penile sizes in apparently healthy term newborns in Ghana.

## Methods

A cross-sectional prospective study of all apparently well term male newborns delivered at Komfo Anokye Teaching Hospital (KATH) was carried out from May 2014 to September 2014. KATH is a tertiary teaching hospital in Kumasi, the second largest city in Ghana. Kumasi is a metropolitan city but majority of its inhabitants are Akans of the Ashanti tribe.

A nurse attached to the paediatric endocrine unit was trained to assist the principal investigator with the genital and anthropometric measurements. Two research assistants were trained to assist the two examiners with positioning of the babies and data entry. All apparently well male term newborns with a gestational age of 40 ± 2 weeks and birth weight between 2.5 and 4.0 kg were included. An initial clinical assessment guided by a screening form was done to find out which babies were eligible. Newborns with major congenital anomalies/ dysmorphism, genital anomalies, breech presentation at birth, maternal pregnancy history of hormonal drug use, maternal signs of virilisation during pregnancy, maternal pre-eclampsia or diabetes were all excluded. For eligible babies, the study was explained to the parent(s) and written informed consent was sought for. The baby was then enrolled if the parent(s) consented. Recruitment was done within 48 h after delivery. A complete antenatal history was obtained from the mother and from the hospital records, including a history of ingestion of herbal medicine or prescribed medications. The gestational age was determined by using the last menstrual period and early ultrasound results and confirmed if necessary with Dubowitz/Ballard score [25]. The socio-economic status of the parents was determined using the Oyedeji classification [26]. A complete physical examination of each baby was done and anthropometric measurements taken. All anthropometric measurements were done by a 2-member team, with one person taking the measurements while the other positioned the baby and helped enter the data onto the Case report form.

In a warm environment, the newborns were put in a supine position and the perineum was adequately exposed. The SPL was measured by holding the glans gently between the thumb and forefinger and measuring from the pubic ramus, along the dorsum, to the tip of the glans penis with a disposable wooden spatula. The shaft of the penis was stretched to the point of resistance and the suprapubic fat pad compressed as completely as possible. The foreskin was not included in the measurement. The tip of the glans was marked on the spatula by a pencil. The distance between the tip of the spatula and the marked point was then measured with a measuring tape/ruler. The penile width was determined with a digital Vernier caliper (Resolution 0.01 mm, Accuracy +/−0.02 mm) by measuring the widest diameter along the shaft to the nearest 0.01 mm. All measurements were done twice and the mean value recorded. Majority (72.1%) of the measurements was done by the principal investigator but fortnightly, genital measurements were done on 5 randomly selected babies by both examiners to evaluate for inter-observer variability, which remained insignificant throughout the study. The standard deviations for inter-observer variation were 0.04 cm and 0.13 mm and 95% of paired measurements were within range of 0.09 cm and 0.26 mm for penile length and penile width respectively.

The data was analyzed using R statistical software version 3.1.2. Continuous variables were summarized and presented as means with standard deviation as well as median with their corresponding ranges. Single categorical variables were tabulated and expressed as percentages. The relationships between the genital measurements and the various categorical variables were determined using an analysis of variance, correcting for possible confounders and reporting the respective *p*-values as appropriate. Relationships between the genital measurement and other continuous variables were however determined using linear regression. These analyses were presented as their regression coefficient with their 95% confidence intervals. To establish the various percentiles for the genital measurements they were each summarized for gestational age to display the; 1^st^, 3^rd^, 50^th^, 97^th^, and the 99^th^ age specific percentiles with the range. For all analysis a two sided *p*-value of <0.05 was considered statistically significant.

The study was approved by the Committee on Human Research, Publications and Ethics of KATH/Kwame Nkrumah University of Science and Technology, Kumasi. The Heads of the Department of Child Health and the Obstetrics and Gynaecology department as well as other staff in both departments were briefed about the study. A written Informed Consent Form detailing the study purpose, benefit and possible risk of the study to the children were provided and explained to the parent(s) in a language they could understand. The child was only enrolled if the parent(s) consented.

## Results

A total of 644 male infants were studied. A summary data of study subjects is shown in Table 1. The distribution of maternal tribe was Akan 451 (70%), Mole-Dagomba 92 (14.3%), Ewe 20 (3.1%), Ga-Adangbe 7 (1.1%) and ‘Others’ (including Hausa, Grushie and Wangara tribes) 74 (11.5%). Genital measurements from study subjects were used to construct gestation-specific genital anthropometry percentile charts (Table 2). The mean penile length (MPL) was 3.3 cm and the −2.5 SD and +2.5 SD for MPL were 2.1 cm and 4.4 cm respectively.

**Table 1:** Summary data of study subjects

	Mean	SD	Median	Minimum	Maximum
Gestational age *(weeks)*	40.0	1.2	40.1	38.0	42.0
Post-natal age *(hours)*	10.1	8.3	8.0	1	48
Five Minute APGAR score	8.6	0.6	9.0	6	10
Birth weight (*kgs*)	3.2	0.4	3.2	2.5	4.0
Length *(cm)*	48.7	2.1	49.0	39.5	54.0
Head Circumference *(cm)*	34.4	1.3	34.4	30.0	38.2
Penile Length (*cm*)	3.3	0.5	3.2	2.0	4.8
Penile Width(*cm*)	1.05	0.1	1.05	0.69	1.37
Heart Rate (*bpm*)	134	11	136	96	164
Respiratory Rate (*cpm*)	49	11	48	28	90
Temperature (*°C*)	35.9	0.8	36	34.0	37.9
Maternal age *(years)*	28.5	6.2	28.0	15	49
Antenatal Care attendance	7	3	7	0	20
Paternal Age (*years*)	34.7	7.4	34	18	65
Parental socioeconomic score	3.3	1.0	3.5	1	5

**Table 2:** Percentile chart for penile size by gestational age in male newborns

Gestational age *(weeks)*	Percentiles						
	Min	1^st^	3^rd^	50^th^	97^th^	99^th^	Max
Penile Length (*cm*)							
38	2.3	2.3	2.6	3.1	4.2	4.3	4.4
39	2.2	2.2	2.5	3.2	4.1	4.3	4.7
40	2.0	2.2	2.2	3.3	4.4	4.5	4.8
41	2.0	2.2	2.3	3.2	4.0	4.3	4.5
42	2.3	2.5	2.7	3.2	4.0	4.1	4.1
Penile Width (*mm*)							
38	7.6	7.7	8.2	10.3	13.1	13.2	13.2
39	6.9	7.4	8.2	10.6	12.8	13.4	13.7
40	6.9	7.7	8.5	10.5	12.5	12.7	13.5
41	7.8	7.9	8.3	10.5	12.4	12.7	13.2
42	7.3	7.5	8.0	10.9	12.3	13.1	13.7

There was no significant difference in genital size by maternal tribe, parental socioeconomic score or maternal herbal intake. Eighty-three mothers (12.9%) took herbs at varying periods during their pregnancy. There was also no significant difference in genital measurements done in the first 24 h of postnatal life and those done from 24 h up to 48 h. MPL, but not mean penile width (MPW), was negatively correlated with maternal age (Correlation Coefficient −0.062, 95% CI −0.121 to −0.002; *p* = 0.04) but positively correlated with gestational age (Correlation Coefficient 0.33, 95% CI: 0.02–0.64, *p*-value = 0.04). In an analysis of variance, only MPL was significantly different by mode of delivery. A post hoc analysis found a difference between only the caesarean section (C/S) group and the spontaneous vaginal delivery (SVD) group (*p* = 0.038). Table 3 shows the correlation between genital size and anthropometric parameters.

**Table 3:** Association of genital size with anthropometric parameters

	Birth Weight (*kgs*)		Length (*cm*)	
	*Coeff (95% CI)*	*P-value*	*Coeff (95% CI)*	*P-value*
MPL *(mm)*	0.87 (−0.1–1.85)	0.08	0.34 (0.16–0.51)	***<0.001***
MPW *(mm)*	0.57 (0.32–0.81)	***<0.001***	0.11 (0.07–0.16)	***<0.001***

*Significant p-values are in bold face*

## Discussion

Micropenis result from a heterogenous group of disorders; the most common being fetal testosterone deficiency [18]. In the human male fetus, testosterone synthesis by the fetal Leydig cell during the critical period of male differentiation (8–12weeks) is under the influence of placental human chorionic gonadotrophin. After mid-gestation, fetal pituitary Luteinizing hormone modulates fetal testosterone synthesis by the Leydig cell and, consequently, affects the growth of the differentiated penis [7]. Growth hormone acting in conjunction with insulin-like growth factors may also modulate androgen action [27]. Thus, males with congenital hypopituitarism as well as isolated gonadotropin or growth hormone deficiency can present with normal male differentiation and micropenis at birth. Rarely micropenis is associated with 5 alpha reductase enzyme deficiency or mild defects in the androgen receptor [7], though patients with 5 alpha reductase enzyme deficiency are more likely to have associated hypospadias than only isolated micropenis [28].

The main purpose of this study was to define normative genital sizes in the Ghanaian male newborn. To the best of our knowledge, this is the first published study of penile size in Ghanaian newborns. The relatively larger sample size allowed for a more precise construction of gestational specific percentile charts than in previously published smaller scale studies. Studies done in different parts of the world have reported varying MPL in newborns ranging from 2.35 to 3.77 cm [3, 14–16, 20, 29, 30]. This study found an MPL of 3.3 ± 0.5 cm. Using the conventional definition of micropenis as SPL < 2.5 SD below the mean and ‘macropenis’ as an SPL > 2.5 SD, a Ghanaian term newborn may warrant investigation if he has an MPL < 2.1 cm or > 4.4 cm.

Among studies with similar methodology, Akin et al. [21] reported a relatively lower MPL of 3.16 ± 0.39 cm in Turkey and Semiz et al. [30] also reported an even lower MPL of 2.81 ± 0.32 cm in 746 term Turkish newborns. Possible factors accounting for this observed difference are inter-observer variation and differences in the characteristics of the study populations including ethnicity/race [17]. Reports from other parts of Asia have also been relatively lower [23, 31], though not uniformly so [18, 22, 32]. Very low SPL have especially been reported in Indonesian newborns [33, 34]. Faizi et al. [29] for example reported a relatively low MPL of 2.35 ± 0.39 cm in 197 term neonates in Indonesia. Few African studies have been published. Jarrett et al. [24] in Nigeria reported a slightly higher MPL of 3.4 ± 0.48 cm. However, the characteristics of their study population were quite different. The study included preterm, term and post term babies, whilst only term babies were analyzed in the current study. Elsewhere in Africa, Kholy et al. [35] also reported a marginally higher MPL of 3.4 ± 0.37 cm in 150 Egyptian neonates. In Caucasians, Schonfeld et al. [14] published a relatively higher MPL in full-term infant of 3.75 ± 0 cm, while Feldman and Smith [3] and Flatau et al. [16] published an MPL of 3.5 ± 0.7 cm and 3.5 ± 0.4 cm, respectively.

Fewer studies have reported penile width in published literature. This study found an MPW of 1.05 ± 0.1 cm. Semiz et al. [30] reported a comparable MPW of 1.04 ± 0.09 while Akin et al. [21] reported a comparatively higher MPW of 1.21 ± 0.11 cm. The technique for the measurement of penile width was not stated in the former whilst the latter study measured the penile width in the midshaft using a circular scale with measurements being taken to the nearest even 2 mm. Jarrett et al. [24] reported a relatively higher MPW of 1.2 ± 0.14 cm. Here the penile width was measured as the transverse diameter in the middle of the penile shaft using a ruler. This current study measured penile width in its widest diameter along the shaft with a caliper which measures to the nearest 0.01 mm. Differences in measurement techniques and accuracy of the scales employed could play a role in the variation of published MPW values. There have also been reports of a significant association between penile width and other anthropometric parameters as well as maternal age [21, 24, 36]. Consequently, differences in the characteristics of the study population may contribute to the variability seen in reported values of MPW. Elsewhere, Feldman and Smith [3] has reported a comparable MPW of 1.1 ± 0.2 cm in the United States whereas Sutan-Assin et al. [33] reported a relatively low MPW of 0.82 ± 0.33 cm in 336 term babies in Indonesia.

Genital size did not vary significantly by parental socio-economic status, probably as a result of the lack of association between anthropometric parameters and parental socio-economic status. The effect of socioeconomic status on genital sizes is likely an indirect one, through an effect on newborn anthropometric parameters. No study has examined the effect of socio-economic status on genital sizes per say. However, Lee et al. [19] reported a significant increase in SPL in Korean children when they compared normative data obtained in the year 2011 to that of 1987. They attributed this to a comparative increase in anthropometric indices like weight and length/height from improved economic conditions. Genital size also did not vary significantly with maternal use of herbal medicine during pregnancy. This analysis was done to evaluate for possible hormonal effects of the ingested herbal medicines, since their constituents were largely unknown. MPW was higher (1.15 ± 0.77 cm) in babies of mothers from the Ga-Adangbe tribe but their numbers were too small to compute for statistical significance. There was no significant difference between genital measurements done in the first 24 h and those done afterwards. Contrarily, in 63 Japanese term newborns Matsuo et al. [31] found that penile length measured within 12 h after birth differed significantly (0.3 cm 95% CI 0.22–0.34) from values obtained 1–7 days postnatally by the same examiner. They attributed this to the oedematous prepubic skin hindering sufficient stretching of the penis. However, the sample size was probably too small to establish a definite significance and *p*-values were not given.

There was a weak inverse relationship between maternal age and MPL (Coefficient −0.062, 95% CI −0.121 to −0.002; *p* = 0.04), consistent with what was reported by Romano-Riquer et al. [36]. The association between penile size and maternal age could be related to the finding that older pregnant women tend to have lower testosterone levels [37, 38], and there is a positive correlation between maternal and fetal testosterone levels [39]. Testosterone has a trophic effect on the phallus, as demonstrated by Mohamed et al. [40] who reported a positive correlation between penile length and serum testosterone levels in newborns. Another possibility is that placental aromatase activity, which metabolizes fetal androgens into estriol, may increase with increasing maternal age, thereby resulting in lower fetal testosterone concentrations. Kaijser et al. [41] however found no correlation between maternal age and placental aromatase.

This study found a significant positive correlation between MPL and gestational age (*p* = 0.04). In multivariate analysis, likely because of the small range of gestational ages (only term neonates delivered at 38–42 weeks of gestation were included); this correlation was no longer statistically significant. Other studies have also reported a positive correlation between MPL and gestational age [23, 34]. Jarrett et al. [24] found no correlation between MPL and gestational age. The reasons for these inconsistencies in finding associations are most likely multi-factorial, possibly including differences in study methodology and ethnicity [17]. Similar to Jarrett et al. [24], this study did not find any correlation between MPW and gestational age. In a multiple linear regression analysis with post hoc analysis, babies delivered by SVD had a significantly higher MPL than those delivered by C/S (*p* = 0.038). We performed this analysis to find out whether the inclusion of babies delivered by other means apart from SVD can influence mean genital size. Kutlu [20] in Turkey recruited only babies delivered by SVD, but most other published studies did not state the mode of delivery [17, 21, 22, 24, 30, 32, 42]. The reason for our finding was not clear. More studies need to be done to clarify this. Our study specifically excluded babies with breech presentation because of its known association with hypopituitarism. Debate is ongoing about whether breech presentation is the cause or consequence of hypopituitarism [43]. Hypopituitarism has also been associated with instrumental delivery [5].

In this study, MPL was positively correlated with length (*p* < 0.001) but not with birth weight (*p* = 0.08) while MPW was proportionally correlated with both anthropometric parameters (*p* < 0.001 for all). These observations closely mirror those of Akin et al. [21]. Likewise, other authors have reported a positive correlation between MPL and birth length [20, 23, 30, 34, 44], although majority also found a positive correlation with birth weight as well, unlike this study [23, 30, 34, 44]. Jarrett et al. [24] and Cheng & Chanoine [17] found no correlation between MPL and birth weight or length. Jarrett et al. [24] also found a positive correlation between MPW and birth weight and length. In contrast, Cheng and Chanoine [17] found no significant correlation between MPW and birth weight or length.

Whether penile length at birth correlates with penile length in adults is unclear. There is to our knowledge no longitudinal study on this topic and no studies directly comparing penile lengths in neonates and adults of diverse origins. Penile length seems to be relatively similar in both neonates and adults from Nigerian and Caucasian descent [14, 24, 45].

## Conclusions

In comparing penile size across studies, characteristics of the study population including gestational age, birth weight and length, mode of delivery and maternal age should be taken into consideration. More importantly, over/under-diagnosis of micropenis may occur with its attendant unnecessary investigations and treatment if local normative values for penile size are not established.

## Limitations

Measurements were done by two examiners and could have introduced an error due to inter-observer variability. However, an extensive training was done and a subset of study subjects was examined by both 2 examiners to evaluate and control for variability, which remained insignificant throughout the study.

## Additional file

Additional file 1: Penile length Ghana. (TXT 75 kb)

## Abbreviations

CI: Confidence interval
Coeff: Correlation coefficient
KATH: Komfo Anokye Teaching Hospital
MPL: Mean penile length
MPW: Mean penile width
SPL: Stretched penile length
